# Improved outcomes from HIV/TB co-infection in Singapore following a switch to earlier anti-retroviral therapy

**DOI:** 10.7448/IAS.17.4.19624

**Published:** 2014-11-02

**Authors:** Barnaby Young, Kalisvar Marimuthu, Gan Suay Hong, Leo Yee Sin

**Affiliations:** 1 Infectious Diseases, Tan Tock Seng Hospital, Singapore, Singapore; 2 Respiratory Medicine, Tan Tock Seng Hospital, Singapore, Singapore

## Abstract

**Introduction:**

Recent clinical trials have provided clear evidence to support early anti-retroviral therapy (ART) in patients with HIV/TB co-infection and low CD4 counts. We investigated how this has changed treatment and outcomes in Singapore.

**Materials and Methods:**

A retrospective review was performed with inpatient and outpatient records for all subjects diagnosed with HIV/TB co-infection from 2006 to 2011 attending the Tuberculosis Control Unit, Tan Tock Seng Hospital, Singapore. Data for subjects with a presenting CD4<200 cell/mm^3^ were extracted and split into two groups, “Delayed”: ART more than 8/52 after starting TB treatment, and “Early”: ART within 8/52 of starting TB treatment.

**Results:**

One hundred thirty-four out of 180 subjects in the database met the inclusion criteria for this study, 89 in the delayed group and 45 in the early. No statistically significant differences in baseline demographics between the two groups were identified. Both groups presented with markedly low CD4 counts, with overall 60% <50cells/mm^3^. Median CD4 counts were lower in the delayed ART group (37 vs 50, p=0.015). Prevalence of other opportunistic infections at TB diagnosis was not significantly different (20%), but TB in the early ART group was more likely to include extra-pulmonary disease (46% vs 57%, p=0.038). Four cases were culture negative, 2 multi-drug resistant and 10 (7.8%) were isoniazid mono-resistant. There was a significant trend to earlier ART with more recent TB diagnosis (p<0.001). In the first 365 days after TB diagnosis, 11 deaths occurred in the delayed ART group, and 0 in the early (p=0.033). A Kaplan-Meier time-to-event analysis demonstrated a clear separation in the frequency of death or opportunistic infections at eight weeks (Figure 1, p<0.001). Immune reconstitution disease was significantly more likely in the early ART group, but did not result in death (9% vs 38%, p<0.001). Treatment interruptions due to adverse drug events (ADE) developed in a median of 25 days (IQR 15–43). Interestingly, early ART was associated with a significantly lower number of treatment interruptions attributed to ADEs, with a higher proportion of patients completing two months of pyrazinamide induction (66% vs 85%, p=0.054) and rifampicin consolidation (79% vs 95%, p=0.03) – after excluding resistance or death. A trend to longer duration TB treatment was observed with delayed ART.

**Conclusions:**

Significant improvements in HIV/TB infection outcomes correlate with the switch to earlier ART.

**Figure 1:**
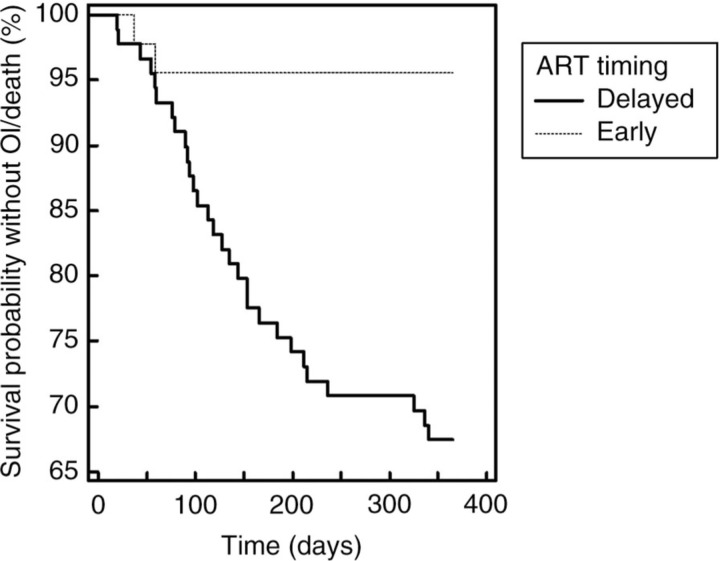
Kaplan-Meier survival curve, censored at first event per subject. Hazard ratio for death or opportunistic infection 8.36 in the delayed ART group (CI 95: 4.02–17.36).

